# Prevalence and genetic variability of *Anaplasma phagocytophilum* in wild rodents from the Italian alps

**DOI:** 10.1186/s13071-017-2221-6

**Published:** 2017-06-14

**Authors:** Fausta Rosso, Valentina Tagliapietra, Ivana Baráková, Marketa Derdáková, Adam Konečný, Heidi Christine Hauffe, Annapaola Rizzoli

**Affiliations:** 1 Fondazione Edmund Mach, Research and Innovation Centre, San Michele all’Adige, TN Italy; 2 Slovak Academy of Science, Bratislava, Slovakia; 3 Masaryk University, Brno, Czech Republic

**Keywords:** *Anaplasma phagocytophilum*, Ear biopsy, *msp4*, *groEL*, *Myodes glareolus*, *Apodemus flavicollis*, Phylogenetic analysis

## Abstract

**Background:**

Human granulocytic anaplasmosis is a zoonotic bacterial disease with increasing relevance for public health in Europe. The understanding of its sylvatic cycle and identification of competent reservoir hosts are essential for improving disease risk models and planning preventative measures.

**Results:**

In 2012 we collected single ear biopsy punches from 964 live-trapped rodents in the Province of Trento, Italy. Genetic screening for *Anaplasma phagocytophilum* (AP) was carried out by PCR amplification of a fragment of the 16S rRNA gene. Fifty-two (5.4%) samples tested positive: 49/245 (20%) from the bank vole (*Myodes glareolus*) and 3/685 (0.4%) samples collected from the yellow-necked mouse (*Apodemus flavicollis*). From these 52 positive samples, we generated 38 *groEL* and 39 *msp4* sequences. Phylogenetic analysis confirmed the existence of a distinct rodent strain of AP.

**Conclusions:**

Our results confirm the circulation of a specific strain of AP in rodents in our study area; moreover, they provide further evidence of the marginal role of *A. flavicollis* compared to *M. glareolus* as a reservoir host for this pathogen.

## Background

*Anaplasma phagocytophilum* (AP) is a gram-negative bacterium transmitted by ixodid ticks, mainly by *Ixodes ricinus* in Europe [1, 2], although *I. tranguliceps* may play a critical role in the sylvatic cycle [3]. This zoonotic pathogen, reported from about 100 species of vertebrates worldwide [4], is responsible for anaplasmosis in livestock and companion animals and therefore is of recognised veterinary relevance. AP also causes human granulocytic anaplasmosis (HGA), an emerging human disease of public health concern [5, 6]. AP primarily invades and replicates in polymorphonuclear leucocytes and usually causes influenza-like symptoms in humans, although infection is occasionally fatal [7]. The epidemiological cycle of this pathogen in Europe is poorly understood but is likely complex given a large number of possible reservoir hosts, the broad distribution and extensive niches of tick vector species and the various bacterial genotypes identified [6].

In Italy, as in the rest of Europe, the majority of studies on this pathogen have focused on the screening of the main tick vector, *I. ricinus*, with prevalence in engorged ticks ranging from 1 to 20% in Europe (see [4] and references therein) and from 4.4% (Province of Belluno [8]) to 24.4% (Region of Lazio [9]). Other European studies have confirmed AP infections in medium-sized and large wild mammals, such as roe deer, red deer, wild boar and red fox [2, 10] as well as in companion animals, like dogs, horses and domestic ruminants [4, 11, 12]. Very little is known about the role of passerine or migratory birds as competent reservoirs or as tick dispersers [13, 14], although Jahfari et al. [1] recently reported that AP genotype IV tends to be associated with bird species. This genotype has not been detected in other vertebrates or questing *I. ricinus* ticks [1].

Here we focus on the role of small mammals, which are well-known reservoirs of many tick-borne pathogens, but also crucial feeding hosts for various stages of *I. ricinus* [15]. Recent observations from central and western Europe, including Italy, suggest that small mammals may have independent epidemiological cycles involving genetically distinct, non-pathogenic AP genotypes [16–18]. In this study we use a collection of tissue samples from live-captured animals in four study sites at two different altitudes in the Province of Trento, Italy, to investigate AP genotypes circulating in rodents in more detail. A large number of PCR positive samples allowed us to confirm the existence of distinct AP strains associated with rodents. Moreover, we extended the knowledge on AP prevalence in rodents in this area.

## Results

Four rodent species belonging to *A. flavicollis* (*n* = 685), *M. glareolus* (*n* = 245), *A. sylvaticus* (*n* = 28) and *Microtus multiplex* (*n* = 6) were captured. Ear biopsies from all 964 individuals were screened for AP. Fifty-two rodents tested positive with an overall PCR prevalence of 5.4% (52/964, 95% CI: 3.97–6.83%); ranging from 0% for *A. sylaticus* and *M. multiplex* to 0.4% (3/685; 95% CI: 0.07–0.87%) for *A. flavicollis* and 20% (49/245; 95% CI: 9–31%) for *M. glareolus*. The difference in prevalence among the two most represented species, *A. flavicollis* and *M. glareolus*, was statistically significant (*χ* ^2^ = 130.82, *df* = 1, *P* < 0.0001), with more PCR-positive bank voles than yellow-necked mice. Only rodents trapped at high altitude study sites were positive for AP (Table 1). The ratio of captured rodent species also differed greatly at the two altitudinal levels with 301 *A. flavicolllis* and 3 *M. glareolus* at the lower altitude sites (100 to 1), and 384 and 242, respectively, at the higher altitude sites (1.5 to 1). AP prevalence between species in the two positive sites was compared, but no statistical differences were observed (Fisher’s exact test, *P* > 0.01).

**Table 1:** Total and average number of ticks by stage, counted on rodent species in the high and low altitude sites in 2012 (Province of Trento, Italy)

	High altitude sites			Low altitude sites		
Species	Total no. of larvae (Mean)	Total no. of nymphs (Mean)	Total (Mean)	Total no. of larvae (Mean)	Total no. of nymphs (Mean)	Total (Mean)
*Apodemus flavicollis*	383 (0.99)	2 (0.005)	385 (1)	1124 (3.84)	17 (0.06)	1141 (3.89)
*Myodes glareolus*	209 (0.87)	10 (0.04)	219 (0.91)	1 (0.33)	0	1 (0.33)
Total no. of ticks (Mean)			604 (0.97)			1142 (3.86)

A total of 1746 ticks (1717 larvae; 29 nymphs) were counted on rodent hosts (see Table 1). *Apodemus flavicollis* hosted a higher number of ticks (*n* = 1526) compared to *M. glareolus* (*n* = 220) (Fisher’s exact test, *P* < 0.0001). The mean and total number of ticks hosted by rodents was higher at low altitude study sites (mean = 3.86; *n* = 1142) compared to high-altitude sites (mean = 0.97; *n* = 604). The 11 specimens of *I. trianguliceps* ticks were collected from the yellow-necked mouse and the bank vole species and were represented by 1 larva, 6 nymphs and 4 adults.

Two hundred and fifty-one animals out of 930 (27%) from this study were screened for the presence of AP in blood pellets in the previous work [18]. One animal tested positive in both ear and blood samples, while 12 animals previously negative on a blood sample, now tested positive on ear punch sample.

**Fig. 1:**
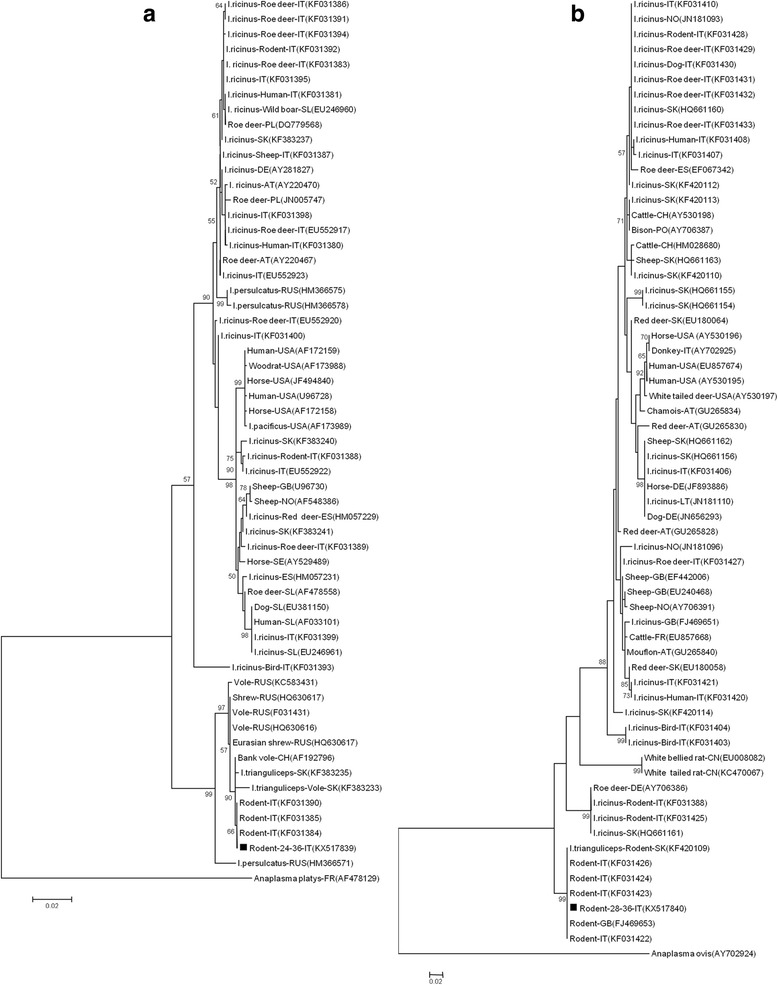
The distance tree inferred by Maximum likelihood analysis using the Tamura-Nei model of 1117 bp long *groEL* gene sequence (**a**) and 298 bp long *msp4* gene sequence (**b**) of *A. phagocytophilum*. Host species, sequences names, two-letter country code and GenBank accession numbers are shown. The numbers at the nodes are bootstrap values expressed as percentages of 1000 bootstrap replicates: the bar (0.02) represents the number of mutations per site. The analysis involved 59 nucleotide sequences of *groEl* and 64 nucleotide sequences of *msp4*. Representative samples of this study are indicated with **▪**

The partial 16S rRNA gene was sequenced for all 52 positive PCR products and confirmed as AP. From these, we also obtained 39 *msp4* and 38 *groEl* sequences. The mean nucleotide diversity (π) among the *A. phagocytophilum* sequences was 0.073 (range 0–0.161) for *msp4* and 0.034 (range 0–0.192) for *groEL*. The maximum likelihood phylogenetic trees for the two partial genes*,* including previously published reference samples [18], and available sequences from the GenBank database, had similar topologies and consisted of two main clades (Fig. 1). The first clade included haplotypes detected in questing *I. ricinus* ticks and various vertebrate hosts such as deer, birds, domestic sheep, domestic dogs and humans from various European countries and the USA. The second clade included only haplotypes from feeding *I. trianguliceps* from Slovakia, as well as rodents and insectivores from various EU countries. Specifically, all *groEL* sequences generated here, regardless of rodent host species, were 100% identical to *groEL* sequences of AP from blood samples of bank voles from Italy (GenBank: KF031390) and 99% to *groEL* sequences of AP extracted from *I. trianguliceps* feeding on voles from Slovakia (GenBank: KF383233, KF383235) [17, 18] (Fig. 1a). The *msp4* representative sequence was 100% identical with that from blood samples of bank voles from Italy (Genbank: KF031422–KF031424, KF031426) and the UK (GenBank: FJ469653), as well as from *I. trianguliceps* feeding on rodents from Slovakia (GenBank: KF420109) [16–18] (Fig. 1b).

## Discussion

Understanding the natural infection cycle of AP is complicated by the presence of several genetic variants carried by some tick vectors feeding on a variety of vertebrate host species. HGA cases are the third most common tick-borne human diseases in USA and Europe [19], although European cases are less severe than Asian and North American ones [20, 21]. Not all AP genetic variants identified are pathogenic to humans, and even if several variants may coexist in the same geographical area, they appear to have distinct enzootic cycles [1, 16, 22]. For example, in the eastern USA, *I. scapularis* hosts both the Ap-V1 non-pathogenic variant as well as Ap-ha pathogenic variant; however, among vertebrates, Ap-V1 is only found in the white-tailed deer (*Odocoileus virginianus*) [23], while Ap-ha only in the white-footed mouse (*Peromyscus leucopus*) [24].

In Europe, several studies have confirmed the importance of *I. ricinus* as a vector of AP [4] and the role of small mammals in maintaining the immature stages of this tick [17, 25, 26]. However, AP infection prevalence in rodents varies considerably between studies and rodent species [4]. A recent phylogenetic analysis suggested that the rodent AP strains belonged to a different cluster to that of other mammals and involved another tick species, *I. trianguliceps* [1]. For this reason, we performed our analysis on a large rodent dataset to improve our knowledge of a possible independent epidemiological cycle for same AP genotype.

In a previous study, we presented an assessment of the circulating genotypes of AP in the rodents in the Province of Trento, using blood samples [18]. The overall prevalence in 1295 animals (*A. flavicollis*, *Moscardinus avellanarius* and *M. glareolus*) was 0.3% (4/1295), but only bank voles were positive (4/100; 4%). In this study, the overall prevalence was 5.4% (52/964). Bank voles were 20% (49/245) PCR-positive and yellow-necked mice 0.4% (3/685). Similar results for AP prevalence in bank voles have been obtained across Europe; for example, 19.2% in Switzerland from blood and tissue samples [25], 3.6–20.5% in Slovakia from ear and spleen samples [17], 13.8–23.1% in France from blood and spleen samples [27] and 22% in Finland from blood samples [28]. To increase the number of bank voles we included sites above 1000 m asl. In fact although this species is well represented in temperate woodlands, it becomes scarce in Mediterranean areas, its southern limit of distribution, where it is influenced by tree cover and height, the presence of dead vegetation, moss and rocks, and moist woodlands [29]. In our study area, these characteristics are satisfied above 1000 m asl where the climate is classified as alpine-continental with an average annual temperature around 8–9 °C and 1000–1500 mm precipitation [30].

In this study, 251 animals screened using ear biopsies were previously PCR-screened from blood pellets in Baráková et al. [18]. Twelve animals testing negative in blood samples tested positive from ear punch samples posing concerns about PCR sensitivity or which sample is better to test. Therefore, possible reasons for these results could be that 1) AP infection in the peripheral blood stream of rodents seems to be short-lived [3, 25, 26, 31, 32]; 2) at the onset of infection the pathogen stays in the peripheral blood before spreading to the overall circulatory system, similar to other tick-borne pathogens [33], but the detection level of AP DNA in blood of non-immune animals reaches and exceeds the threshold of PCR sensitivity only a few days after tick detachment [34]. Since ear biopsies are a less invasive biological sample yet more efficient in the detection of AP compared to blood or organs collection in rodents, we conclude that ear biopsies should be used in the future for screening AP whenever possible.

Our study presents a phylogenetic analysis of the largest number of AP sequences from rodents thus far. The use of specific molecular markers has recently helped to discriminate between genotypes and revealed the existence of an independent epidemiological cycle involving rodents as reservoir hosts, the tick *I. trianguliceps* as a vector [16, 17] and a non-pathogenic AP strain. The *groEL* and *msp4* sequences generated in this study (Fig. 1) confirm that a single rodent AP genotype circulates in Italy, distinct from the strains found in other hosts, as previously suggested by Bown et al. [16] and by Baráková et al. [18]. This genotype is also identical to those reported from the UK in the field vole (*Microtus agrestis*) and bank vole [3], and in Slovakia in the yellow-necked mouse and *I. trianguliceps* [17]*.*

Among all the questing and feeding ticks screened for the presence of AP in our previous study [18], only *I. ricinus* tested positive, and the AP genotype was different from that found in rodents [6, 16–18, 26]. In this study the number of ticks counted on the rodents was higher at low altitudinal level (Table 1), in particular on the yellow-necked mouse. Other authors in Europe reported a higher infestation rate of *I. ricinus* on *Apodemus* species compared to *M. glareolus* [35–38] although some exceptions are noted [39–42]. The role of *I. ricinus* as important vector of rodent AP infections in continental Europe has been described [25], but evidence showed that if only this species is present, AP cannot be maintained in woodland rodent communities, on the contrary, this is achieved where *I. trianguliceps* is also present [3, 26]. In addition, no efficient transmission of AP to *I. ricinus* larvae under laboratory conditions has been confirmed using field-captured tick-infested rodents [43]. Although some authors reported the presence of a small proportion of infected *I. ricinus* larvae, supporting the hypothesis that all three tick stages are involved in AP transmission [1, 32, 44], this mode remains minor and needs to be further investigated. The presence of *I. trianguliceps* has been recently confirmed in northern Italy [18]. *I. trianguliceps*, also known as vole tick, is endophilic, host specialist, non-questing and usually present at very low level on its rodent host [3, 45]; for these reasons chances of contacts with humans are unlikely. Nonetheless, the main concern is that the sympatric presence of both specialised and generalised species has been shown to have a role in maintaining a high level of infection on rodent hosts [1, 3, 46]. In our specific context, *I. trianguliceps* may be important in maintaining high infection levels in the reservoir hosts with regards to *Babesia microti* and *Anaplasma phagocytophilum* [1, 3, 47]. Moreover, due to the complexity of interactions between ticks, vertebrates and associated pathogens, the enzootic infections maintained in an *I. trianguliceps*-rodent cycle could escape into other hosts, including humans [3].

## Conclusions

Using a high number of positive rodent samples and sequenced partial genes than previous studies we have shown that in the Province of Trento, *Anaplasma phagocytophilum* is present and circulates predominantly among rodents, especially bank voles, with a distinct enzootic cycle. The presence of the tick species, *I. trianguliceps*, could not be quantified. The strain of AP found in rodent ear punches in this study was 100% identical to that found in rodents and *I. trianguliceps* reported in other European studies. From an infectious control perspective, it is important to clarify the role of small mammals in the transmission cycle of *Anaplasma phagocytophilum* and the role of other tick species in maintaining high infection levels in the reservoir hosts and acting as possible bridging vectors for the rodent genotype toward humans or domestic animals.

## Methods

### Study sites and rodent sample collection

**Fig. 2:**
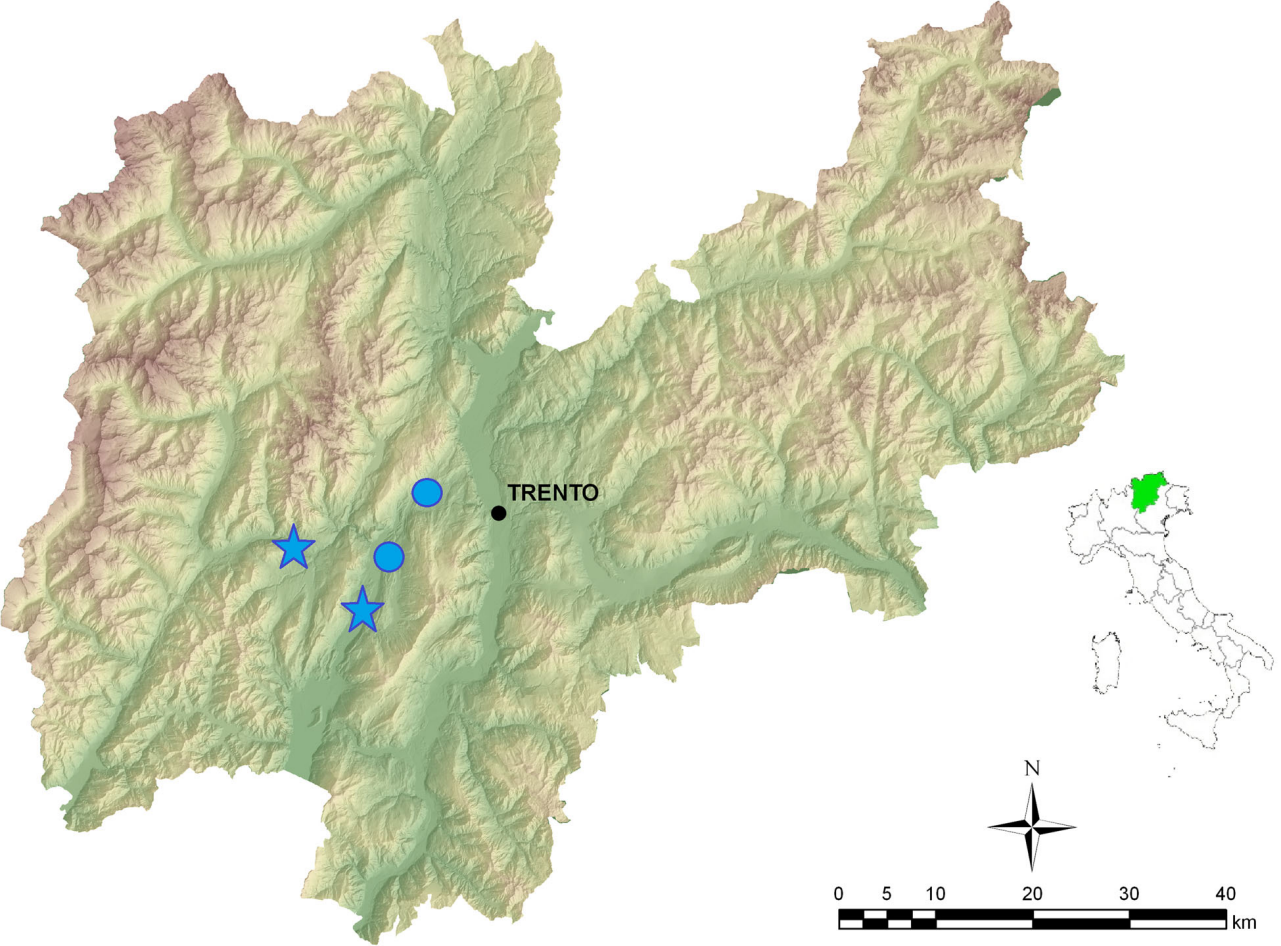
Map of the Province of Trento, Italy, showing the position of the rodent sampling sites (stars indicate high altitude sites; circles indicate low altitude sites). Map is a high-resolution digital elevation model (10 m) and land use map both provided by the “P.A.T. S.I.A.T.” service of the Province of Trento

Small rodents were live-trapped in the Province of Trento, Italy, from April to October 2012 using multiple-capture Ugglan live traps (model 2, Granhab, Sweden), set in 8 × 8 square array grids with a 10 m inter-trap distance. Four grids were set at each of four locations in beech forests where both rodents and ticks are common: two locations were at 700 m above sea level (asl) and two at 1200 m asl (Fig. 2). Each individual was tagged with a subcutaneous passive integrated transponder (pit-tag ID100 Trovan®, UK) to ensure that each individual was identifiable and only sampled once. We routinely counted ticks on hosts, represented mainly by the larval stage, but did not remove them, so the identification with the naked eye at species level was not possible and therefore we cannot assess the proportion of the two feeding tick species. For the presence and identification of *Ixodes* species on rodents, we refer to the previous study described in Baráková et al. [18], where some tick specimens were randomly collected from the animals and genetically identified, contributing to the first record of *I. trianguliceps* in the Province of Trento. An ear biopsy was taken at first capture using sterile disposable ear punch needles (Ø 3 mm), and samples were stored individually and frozen at -80 °C until analysis. Since both *A. flavicollis* and *A. sylvaticus* occur in sympatry in the Province of Trento and are not reliably identifiable by eye, we confirmed that all *Apodemus* captured were *A. flavicollis* following Michaux et al. [48]. Of the 930 animals screened using an ear biopsy in this study, 251 were previously screened for the presence of AP in blood pellets [18].

### *Anaplasma phagocytophilum* detection

DNA was isolated from each ear biopsy using the DNAeasy blood and tissue kit (Qiagen, Hilden, Germany) according to the manufacturer’s protocol. The presence of AP was detected by amplifying 546 bp of the 16S rRNA gene using a nested PCR with primers ge3a/ge10r and ge9f/ge2 after [49]. A reaction without template (water added) served as negative control while AP-DNApositive amplified from *I. ricinus* was used as positive control. Positive PCR products were purified using Pure Link quick gel extraction and PCR purification combo kit (Invitrogen, Thermo Fisher Scientific Baltics, Lithuania) and sequenced using the primers listed above. A BLAST search was performed for all sequences. For AP positive samples, variable partial genes, *msp4* and *gro*EL were amplified and sequenced as already described in [50, 51].

### Statistical and phylogenetic analysis

Fisher’s exact test or Chi-squared tests were carried out to determine if there were statistical differences in the prevalence of AP between the two main rodent hosts and among sites and altitudes.

Nucleotide sequences were verified using Sequencer 4.7, assembled using MEGA6 software and further aligned with ClustalW [52]. The alignment showed that all samples from this study were 100% identical. Therefore only one representative sequence was used for subsequent phylogenetic analysis. MEGA version 6 was used to construct a phylogenetic tree for each gene fragment (Fig. 1) [52]. The maximum-likelihood algorithm [53] with Tamura-Nei model [54] were used with Felsenstein’s [55] bootstrap test of 1000 iterations. The analysis involved 59 nucleotide sequences of *groEl* and 64 nucleotide sequences of *msp4* downloaded from NCBI and 1 representative *gro*EL and *msp4* nucleotide sequence from this study (GenBank Accession nos. KX517839 and KX517840, respectively).

## Abbreviations

AP: *Anaplasma phagocytophilum*
Asl: above sea level
HGA: human granulocytic anaplasmosis
